# Seagrass on the brink: Decline of threatened seagrass *Posidonia australis* continues following protection

**DOI:** 10.1371/journal.pone.0190370

**Published:** 2018-04-06

**Authors:** Suzanna M. Evans, Kingsley J. Griffin, Ray A. J. Blick, Alistair G. B. Poore, Adriana Vergés

**Affiliations:** 1 Evolution and Ecology Research Centre, School of Biological, Earth and Environmental Sciences, University of New South Wales, Sydney, NSW, Australia; 2 Centre for Marine Bio-Innovation, School of Biological, Earth and Environmental Sciences, UNSW Australia, Sydney, NSW, Australia; 3 Sydney Institute of Marine Science, Mosman, NSW, Australia; College of Charleston, UNITED STATES

## Abstract

Seagrasses are in decline globally due to sustained pressure from coastal development, water quality declines and the ongoing threat from climate change. The result of this decline has been a change in coastal productivity, a reduction in critical fisheries habitat and increased erosion. Attempts to slow this decline have included legislative protection of habitat and direct restoration efforts. Monitoring the success of these approaches requires tracking changes in the abundance of seagrasses, but such monitoring is frequently conducted at either too coarse a spatial scale, or too infrequently to adequately detect changes within individual meadows. Here, we used high resolution aerial imagery to quantify the change in meadows dominated by *Posidonia australis* over five years at 14 sites in five estuaries in south-eastern Australia. Australia has some of the world's most diverse and extensive seagrass meadows, but the widely distributed *P*. *australis* has a slow growth rate, recovers poorly after disturbance, and suffers runaway attrition if the conditions for recovery are not met. In 2010, after declines of 12–57% between the 1940s and 1980s, *P*. *australis* was listed as a threatened ecological community in New South Wales. We quantified changes in area at fine spatial scales and, where loss was observed, describe the general patterns of temporal decline within each meadow. Our results demonstrate that seagrass meadows dominated by *P*. *australis* underwent declines of ~ 2–40% total area at 11 out of 14 study sites between 2009 and 2014. In the iconic Sydney Harbour, our analyses suggest that *P*. *australis* meadows are declining at an average rate greater than 10% yr^-1^, exceeding the global rate of seagrass decline. Highlighting these alarming declines across the study region should serve as means to prioritise management action and review the effectiveness of legislative listing as a method to limit impacts at an ecosystem level.

## Introduction

More than a billion people now live within 50 km of the coast worldwide, exerting substantial pressure on critical ecosystems including seagrasses, coral reefs, mangroves and saltmarshes [1–3]. Seagrasses are amongst the most valuable and threatened ecosystems, together with coral reefs and rainforests [4–6], but since 1990, seagrasses have declined globally at the rate of 7% per year [7]. These declines have led to a range of conservation efforts [8] including: extinction risk assessment [3], investment in improved water quality [9,10], redesigned boating infrastructure [11], regulation of damaging fishing activities [12] and direct restoration efforts [13].

The success of seagrass conservation efforts requires monitoring to quantify recovery, if present, and best allocate limited resources to future efforts [14]. Seagrass meadows have been historically mapped using direct field observations, aerial photographs or satellite images. Directly measuring the presence and density of individual seagrass species in the field provides high quality fine-scale data, but the costs associated with this approach tend to be prohibitively high, making this option impractical for mapping large areas. In contrast, inexpensive remote sensing techniques (e.g. satellite imagery) focus on larger scales [15,16] and may therefore miss significant short-term or small spatial changes in seagrass cover [17]. Aerial imagery captured using low-flying aircraft can provide high resolution photographs that can be orthorectified and used to map seagrass at finer scales. This, however, can be an expensive option, particularly when multiple flights are needed to investigate temporal change over both short and long time frames [18]. Development of commercially-available databases of aerial imagery for multiple purposes have since reduced the need for chartering custom flights, reducing the cost of obtaining imagery and enabling the mapping of fine resolution changes in seagrass meadows with less resource investment.

Australian seagrass meadows are some of the most extensive and diverse in the world [19] and are therefore of global importance. Seagrass in Australia covers approximately 50 000 km^2^ [15] and enhances commercial fishery production in southern Australia by AUD 230 000 ha^-1^ year^-1^ [20]. In the last century, natural and anthropogenic damage and subsequent loss of seagrass meadows have accelerated in Australia [21–25], displacing 1450 km^2^ (~2.9%) of seagrass between 1940–1997 [15]. The result has been a concomitant change in coastal productivity, the loss of critical habitat for many species, and increased sediment instability, exacerbating beach erosion [21].

In temperate Australia, the endemic seagrass *Posidonia australis* is one of the most ecologically valuable and structurally complex species [26]. The leaves of *P*. *australis* have a large surface area and deep rhizome matting which support more epiphytes, infauna and associated epifauna than smaller species [15]. Genetic analyses by Evans et al. (2014) suggest that extant *P*. *australis* meadows in southeastern Australia have been in existence since sea levels stabilised ~6000 years ago. Naturally slow-growing, recruits of *P*. *australis* take at least 10 years to mature [27] and centuries to form large (>1km^2^) meadows [25]. It is assumed that *P*. *australis* has a similar lifespan to its Mediterranean relative, *P*. *oceanica*, which has an estimated lifespan of hundreds to thousands of years [28].

In 2010, *Posidonia australis* meadows from six estuaries on the southeastern coast of Australia were formally listed as endangered under the New South Wales Fisheries Management Act 1994, after suffering losses of 12–57% between the 1940s and 1980s [e.g. [22]; [29]; [30]; Glasby2015a]. In 2015, these meadows were subsequently listed as endangered under the Australian Environment Protection and Biodiversity Conservation Act 1999.

Historically, the most common causes of *Posidonia australis* decline in Australia have included major storm events [22], eutrophication from industrial and agricultural run-off [31], smothering by sediment movements [32], dredging and construction [22], and boating impacts (including from moorings, anchors and propellers: [15]; [25]]. Threatened meadows of *P*. *australis* are also generally in areas of high boating activity, where anchors and mooring gear directly impact both individual plants and the structure of rhizome matting [25]. The damage caused by individual boat anchors or moorings might be considered small-scale relative to severe storms or commercial developments [15], but the damage results in a matrix of clearings within the seagrass meadow. Once a *P*. *australis* meadow is fragmented, the sediment trapped beneath becomes vulnerable to erosion, further increasing rates of attrition, and undermining the conditions necessary for growth [33]. In this way, scours destabilise the sediment, interfering with the physical integrity of the meadow and leading to runaway fragmentation across larger areas [25,34]. It has been suggested that once degradation of *P*. *australis* has begun it is a self-perpetuating process [33], and hence the ability of *P*. *australis* to recover from habitat fragmentation is considered extremely low.

In instances where the original cause of seagrass decline has been abated, seagrasses from the genera *Zostera* and *Halophila* are known to re-establish within months to years following disturbance [35,36]. In contrast, larger, slow-growing species such as those from the genus *Posidonia* may take decades or centuries to recolonise disturbed areas [37]. Particularly slow re-colonisation of *P*. *australis* has been recorded in southeastern Australia, where the rate of revegetation of bare patches in damaged areas has been estimated at an average of just 21 +/- 2 cm year^-1^ [37]. In some cases, the seagrass has shown no regrowth in damaged areas even 50 years following disturbance [24,33,37], and without targeted restoration attempts, may never return [15].

The endangered classification of *Posidonia australis* in six estuaries on the southeastern coast of Australia was based on a combination of historical aerial photographs and records from scientific literature [22,29,30,38]. In 2009, Creese et al. mapped seagrass distribution (including *P*. *australis*) across all NSW estuaries, providing the most definitive distribution maps available in the region. However, Creese et al. (2009) also noted that broad-scale mapping across entire estuaries can overestimate seagrass abundance by not recognising smaller-scale, within meadow declines, such as blowouts caused by boat moorings. There is, therefore, a need to develop statistically robust, cost-effective methods of mapping and monitoring *P*. *australis* to identify populations at risk and measure possible recovery.

We quantified the decline of *Posidonia australis* in southeast Australia between 2009 and 2014, using high resolution imagery from Nearmap Australia PhotoMaps [39]. We assessed fourteen sites from five estuaries, to test whether *P*. *australis* has continued to experience loss in recent years following statewide protection (from 2009 to 2014). If loss was observed, we further aimed to describe the general patterns of temporal decline within each meadow.

## Methods

### Study sites

**Fig 1:**
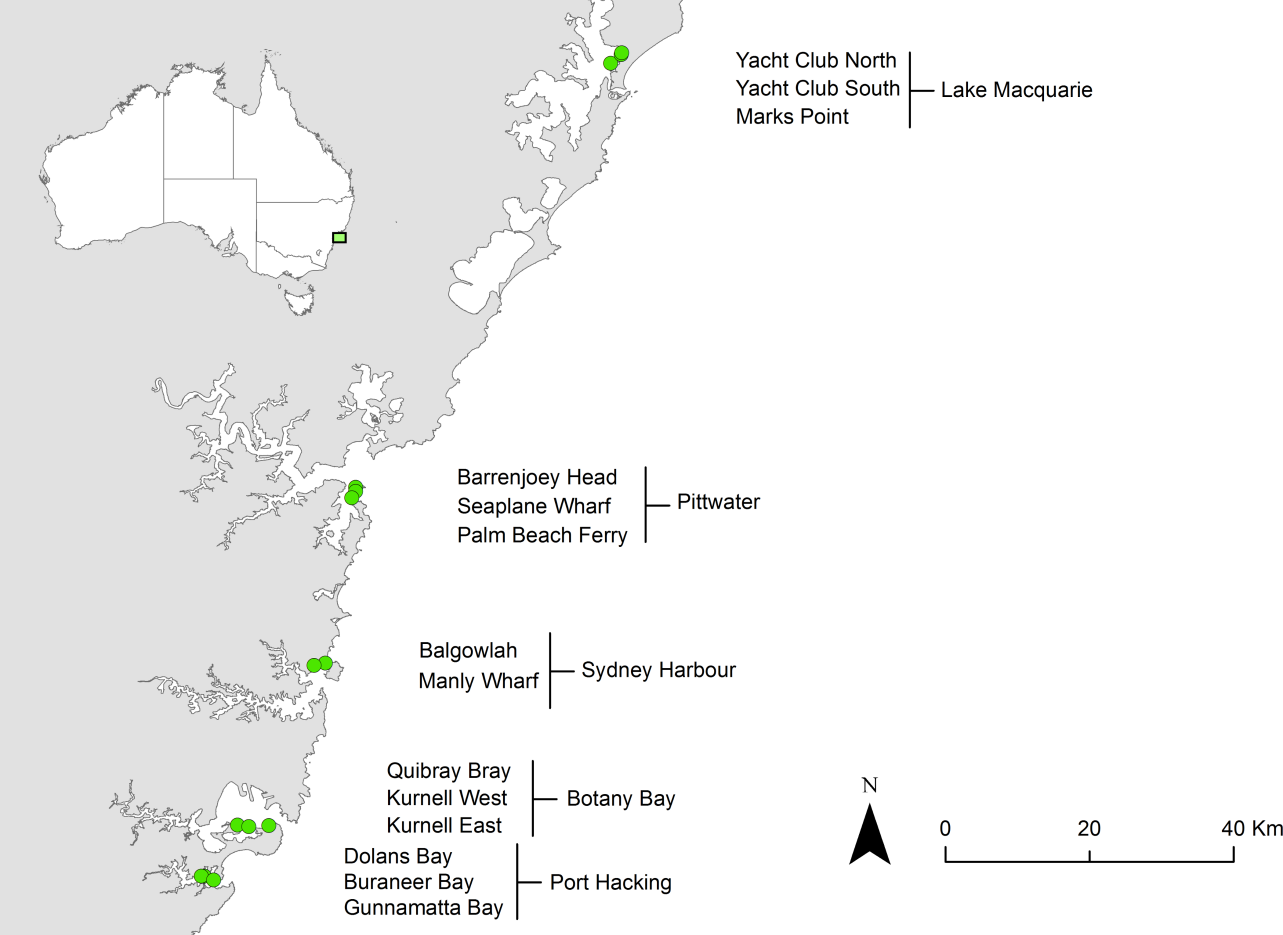
Map of locations along the New South Wales coastline where aerial imagery was obtained to estimate *P*. *australis* declines from 2009–2014. Map boundaries under CC Licence, originally published in 2004 by Geoscience Australia.

To quantify changes in seagrass cover in meadows dominated by *Posidonia australis* between 2009 and 2014, aerial imagery was obtained from Nearmap Australia for three sites within each of five highly impacted estuaries: Lake Macquarie, Pittwater, Sydney Harbour, Botany Bay and Port Hacking (Fig 1). Only two sites were studied in Sydney Harbour due to the lack of an appropriate third site. The total area of *P*. *australis* within these five meadows was estimated at 5.6 km^2^ by [25], making up 25.3% of total seagrass species area across all estuaries on the east coast of Australia. Within these estuaries, *P*. *australis* is mostly restricted to relatively shallow waters (<10 m) with high salinity and low nutrients [40].

### Aerial imagery and mapping source: Nearmap

We used aerial imagery spanning approximately five years (ranging from 20 Oct 2009 to 11 Sep 2014) that were acquired from Nearmap Australia PhotoMapsTM [39]. Available imagery was accessed using the WMS Server loaded in ArcGIS 10.2.2 [41]. The Nearmap database provides an archive of georectified aerial imagery captured at a resolution of approximately 7.5 cm ground sampling distance (GSD). We used a mapping scale of 1:2500 which covered an area of approximately 52 hectares for each site, following the map projection GDA94 MGA Zone 56. Each site contained predominantly *Posidonia australis* habitat, though some images unavoidably contained some rocky substrata or artificial structures (e.g. wharfs). Using the mapping scale to drive the delineation of sites meant that seagrass meadows generally extended past the site 'boundary'. The final site choices were based on field observations that confirmed that the meadows were predominantly made up of *P*. *australis*.

The temporal resolution at each site was largely determined by the proximity of the seagrass meadow to populated areas or infrastructure, where Nearmap tends to collect images more frequently. The number of images finally selected was based on availability, image quality, and clarity of seagrass in the image. This ranged from five (one image each year) to 26 images per site. Change in meadow area was calculated using the first and last available images for each site (methods outlined below), while the full set of available images were used to calculate the rate of decline using a rapid point-sampling method.

In order to attribute seagrass loss to different human impacts, all declines were further investigated using available literature, government documents and council reports. For example, damage caused by boat moorings, anchors and propellers has been reported in these estuaries by West et al. [25], sediment instability in Port Hacking is documented by Albani & Cotis [42], and the laying of submarine electricity cables in Botany Bay was reported by NSW Department of Planning and Infrastructure [30].

### Image classification

**Fig 2:**
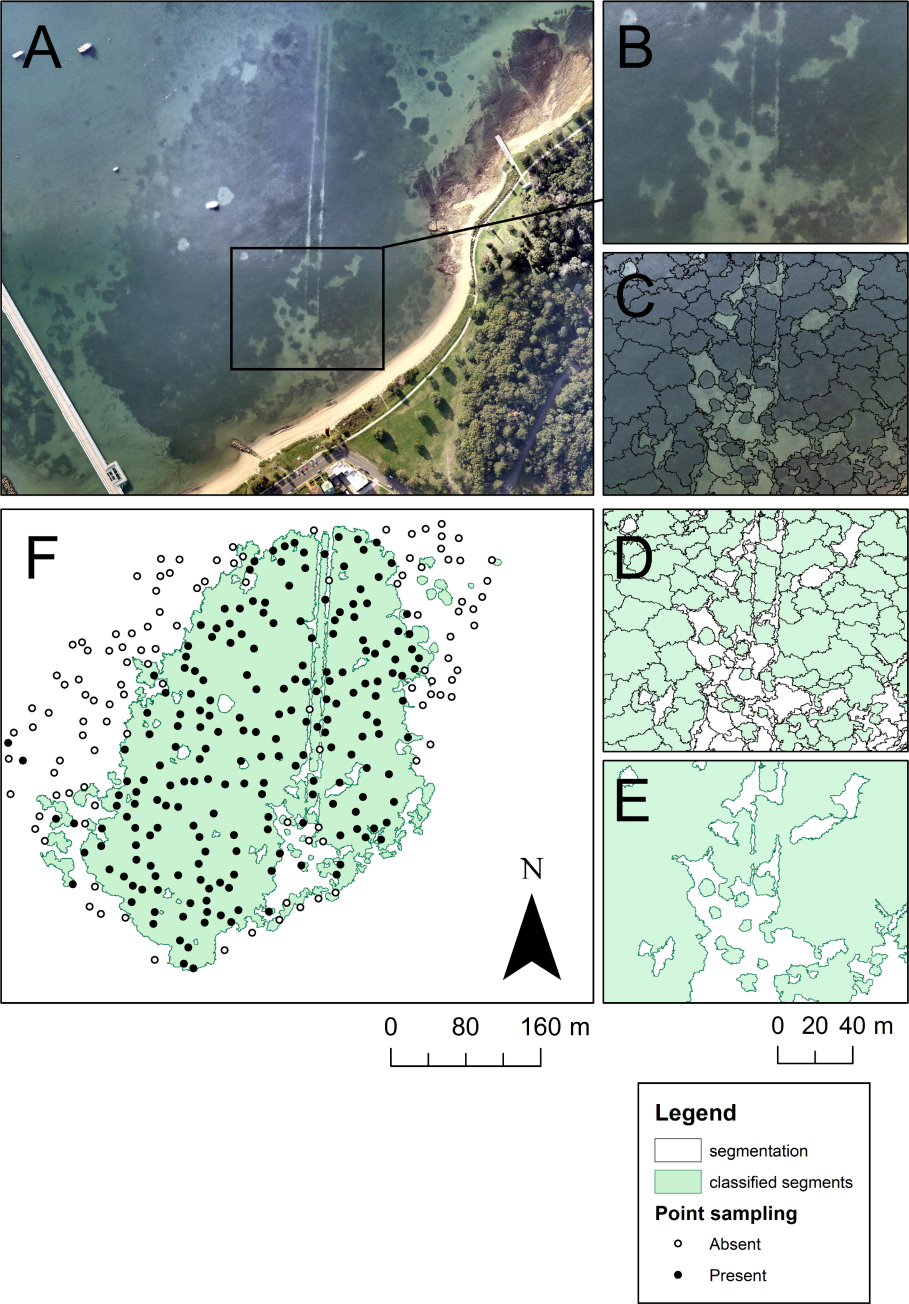
Techniques used to classify *P*. *australis* area using aerial imagery. The example above (A) is the most recent image from Kurnell East in Botany Bay (June 2014, [39]). An example of fragmentation is enhanced (B), showing meadow damage following the laying of submarine cables. A multi-resolution segmentation algorithm was used to segment the image into polygons based on colour and texture (C), which were then manually classified as *P*. *australis* (pale green) or ‘other’ (D). The polygons were then dissolved to represent the full area of *P*. *australis* in the image (E), which could then be formally analysed. Point sampling (F) was used to rapidly estimate percent cover of *P*. *australis* from the remaining time series imagery to determine the rate of change in meadow area. Aerial imagery reprinted under a CC BY license with permission from Nearmap, original copyright 2014.

Two methods were used to quantify the area of seagrass habitat lost over the five year period. A multi-resolution segmentation algorithm was used to convert the aerial imagery from two time points (initial and final) into polygon features (parameters: scale = 0.5, shape = 0.1, compactness = 0.9) based on texture and colour in eCognition Developer 64 version 8.8 [43]. The final number of polygon features per image ranged between 4778 and 15578. This wide variation is a result of the proximity of the seagrass meadow to land-based objects (such as buildings) that substantially increase the number of polygons generated in eCognition. All polygons were then converted to a shapefile and manually classified in ArcGIS (Fig 2). The total area of seagrass quantified using the multi-resolution segmentation algorithm ranged from 4.8 to 25.2 hectares per site.

The chronological changes in meadow extents were quantified at a set of randomly generated points in ArcGIS 10.2.2 [41]. The temporal resolution was largely determined by the quality of photos available from Nearmap; however, in most cases there was at least one useable photo per year. The initial orthophoto classification from the earliest available time point was used to generate a minimum bounding geometry (convex hull) surrounding the extent of the meadow within the image. This resulted in a continuous border around the meadow that contained areas with and without seagrass (e.g. bare sand) that could then be searched using a point sampling framework. 500 points were then randomly generated within the site border, with a minimum between-point separation distance of 10 m, and the locations fixed across time. The number of points at each site scaled to the overall area of the meadow (10m separation equates to approximately 20 points per hectare).

### Ground truthing

All sites were visited at least once per year between 2011 and 2013 to ground-truth the images and ensure that *Posidonia australis* was the dominant marine plant growing in the areas designated as habitat following detection in the imagery. In Sydney Harbour we made an exception—we included the study site at Balgowlah even though it was dominated by *Zostera muelleri* because it was one of just two sites with P. australis present (S5 Fig). For final confirmation, at the conclusion of the study, we visited and photographed the seagrasses at each site, and quantified the relative species cover with a 25 random point-count method using CoralNet software [44] to determine relative cover of *P*. *australis* and other species (see supplementary materials).

### Data analysis

The polygon features were imported into ArcGIS 10.1 and classified manually as either *Posidonia australis*, bare sand, other seagrass (primarily *Zostera muelleri*)/seagrass wrack or shadows/rocks/other. The presence of other seagrass species such as *Z*. *muelleri* was distinguished from *P*. *australis* based on a combination of multiple field observations from 2009–2014 and the growth pattern of *Z*. *muelleri* in this region (primarily restricted to the shallow edges of *P*. *australis* meadows where it can survive intertidal exposure). Known patches of *Z*. *muelleri* in the intertidal zone show up on aerial imagery as a paler, ‘sparse’ brown colour. These patches also wildly fluctuate over short timespans (weeks to months) and are thus quite easily distinguishable from the more stable, slow-growing *P*. *australis* patches.

Due to temporal changes in the appearance of seagrass wrack, water clarity, boat wakes, etc., we classified each polygon by cross referencing with georeferenced images from the same site taken in a similar time frame. Specifically, a polygon was only classified as *P*. *australis* if the same area of meadow was evident in all images within 1–3 months of the image in question. For sites in which only one image per year was useable for the purposes of accurately measuring meadow area, other photos of lesser quality could be used to confirm the presence of seagrass in areas that were otherwise temporarily obstructed (e.g. in the shadow of a moored boat).

**Fig 3:**
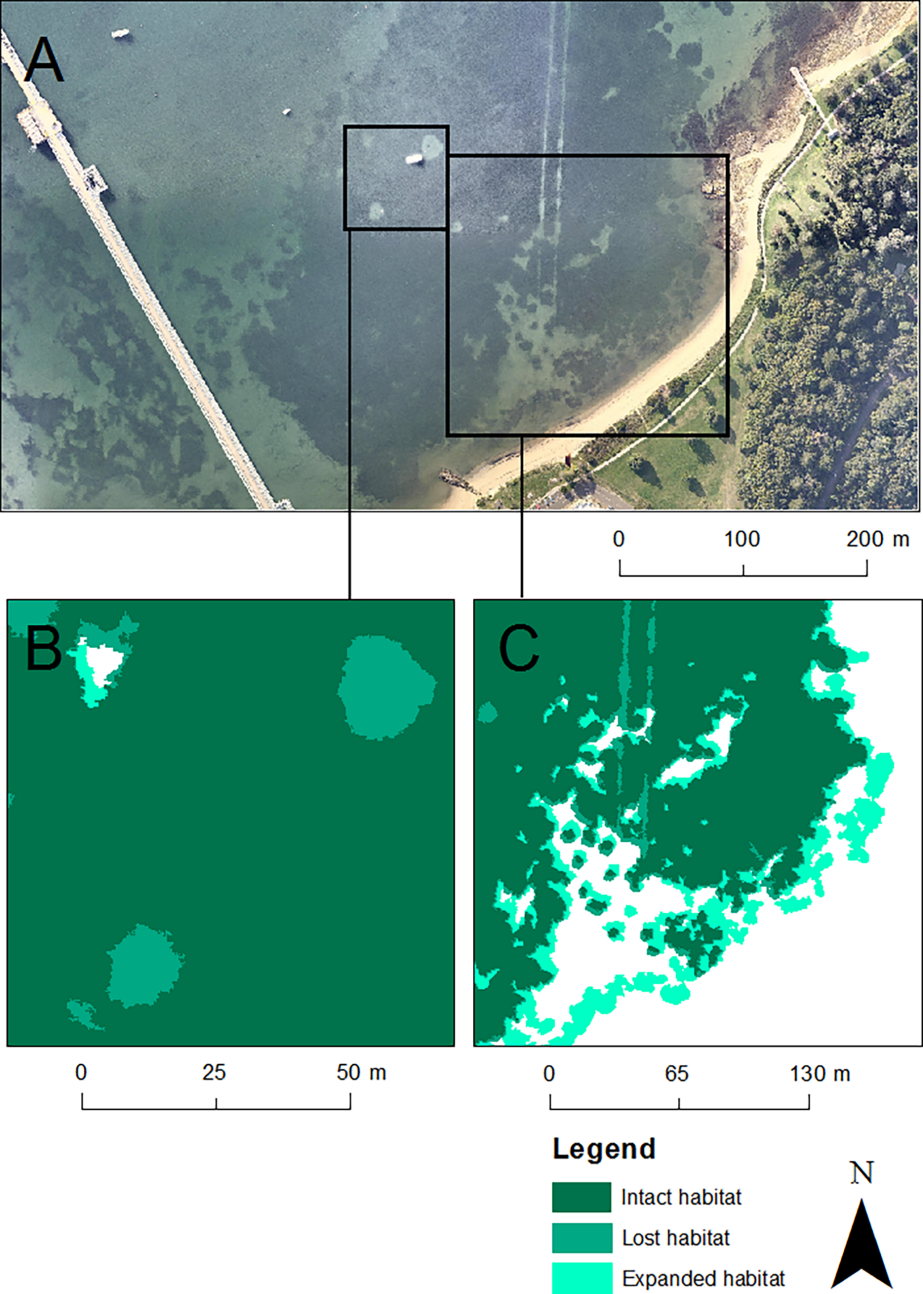
Visual representation of change in meadow area. The image of Kurnell East in Botany Bay (June 2014, [39]) is represented by (A). Inlaid boxes (B) and (C) represent the extent of meadow area both lost and gained since April 2010. Light brown = intact meadow; dark brown = meadow area lost since 2010; pale yellow = meadow area gained since 2010. Aerial imagery reprinted under a CC BY license with permission from Nearmap, original copyright 2014.

Following classification, all polygons identified as *P*. *australis* were dissolved (Fig 2) to calculate the overall area and perimeter of individual patches within the meadow. We then compared the full set of measurements from the initial and final images to estimate the dynamic of meadow change over the approximate five year time-frame (illustrated in Fig 3). Using the estimates of percent cover within each image from the point-sampling data (Fig 2F), we calculated the percent change in area of each meadow over time. These values varied slightly from those obtained using polygon classification, which is to be expected given differences in the positioning of random points. However, as the positioning of points did not change across images, we could use this method to estimate the approximate rate of meadow decline. The percentage change in cover from the initial sampling date was analysed as a time series using generalized additive modelling in the package ‘mgvc’ in R [45] with an autoregressive error term to account for temporal autocorrelation and visualised as thin-plate splines. To compare the total percentage loss of *P*. *australis* among the five estuaries over five years, a one-way analysis of variance was performed using sites within estuaries as replicates.

### Results

**Fig 4:**
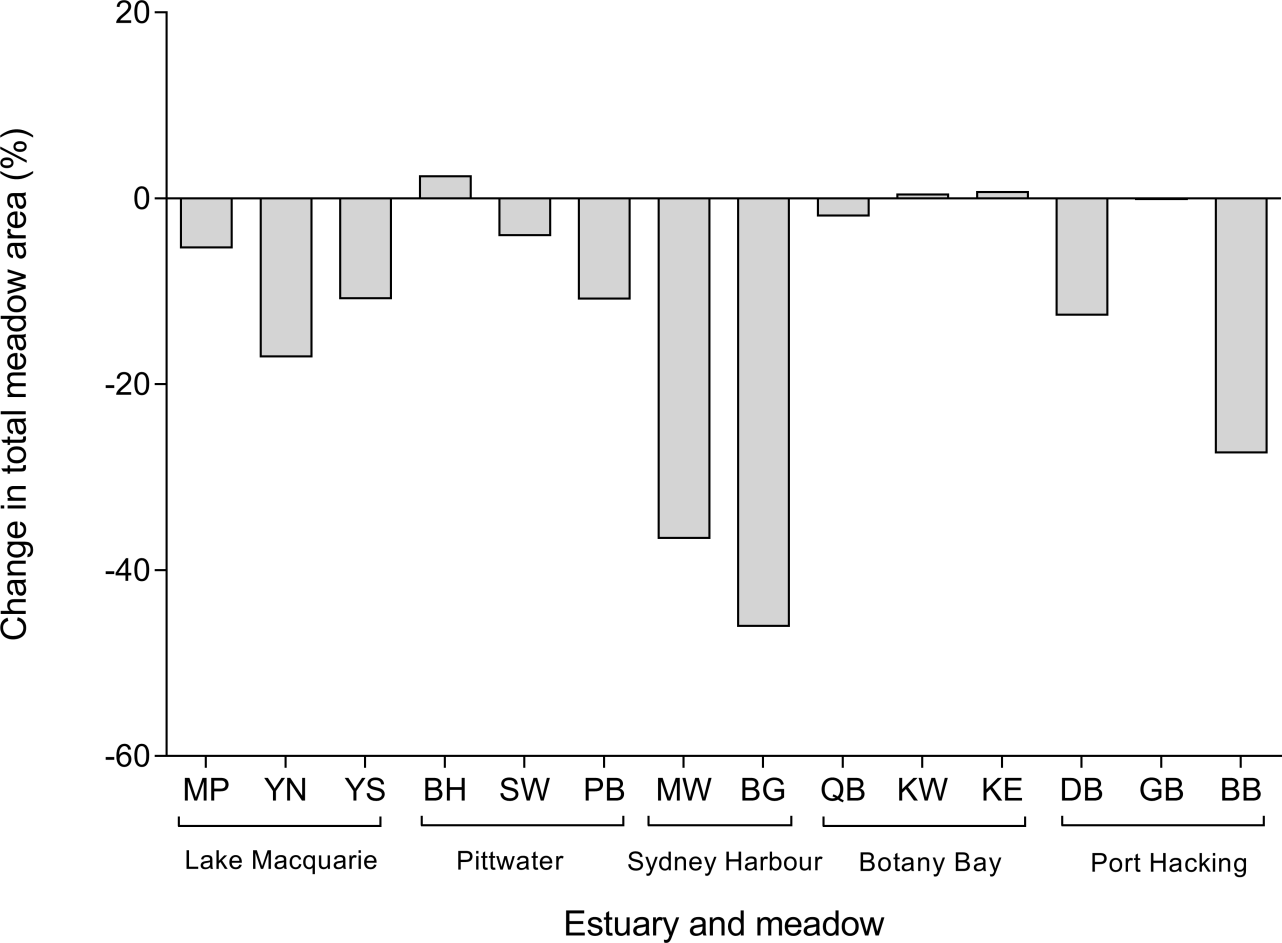
Percent change in meadow area for sites within estuaries of the Manning-Hawkesbury ecoregion from 2009–2014. MP = Marks Point, YN = Yacht Club North, YS = Yacht Club South, BH = Barrenjoey Head, SW = Seaplane Wharf, PB = Palm Beach Ferry, MW = Manly Wharf, BG = Balgowlah, QB = Quibray Bay, KW = Kurnell West, KE = Kurnell East, DB = Dolans Bay, GB = Gunnamatta Bay, BB = Burraneer Bay.

**Fig 5:**
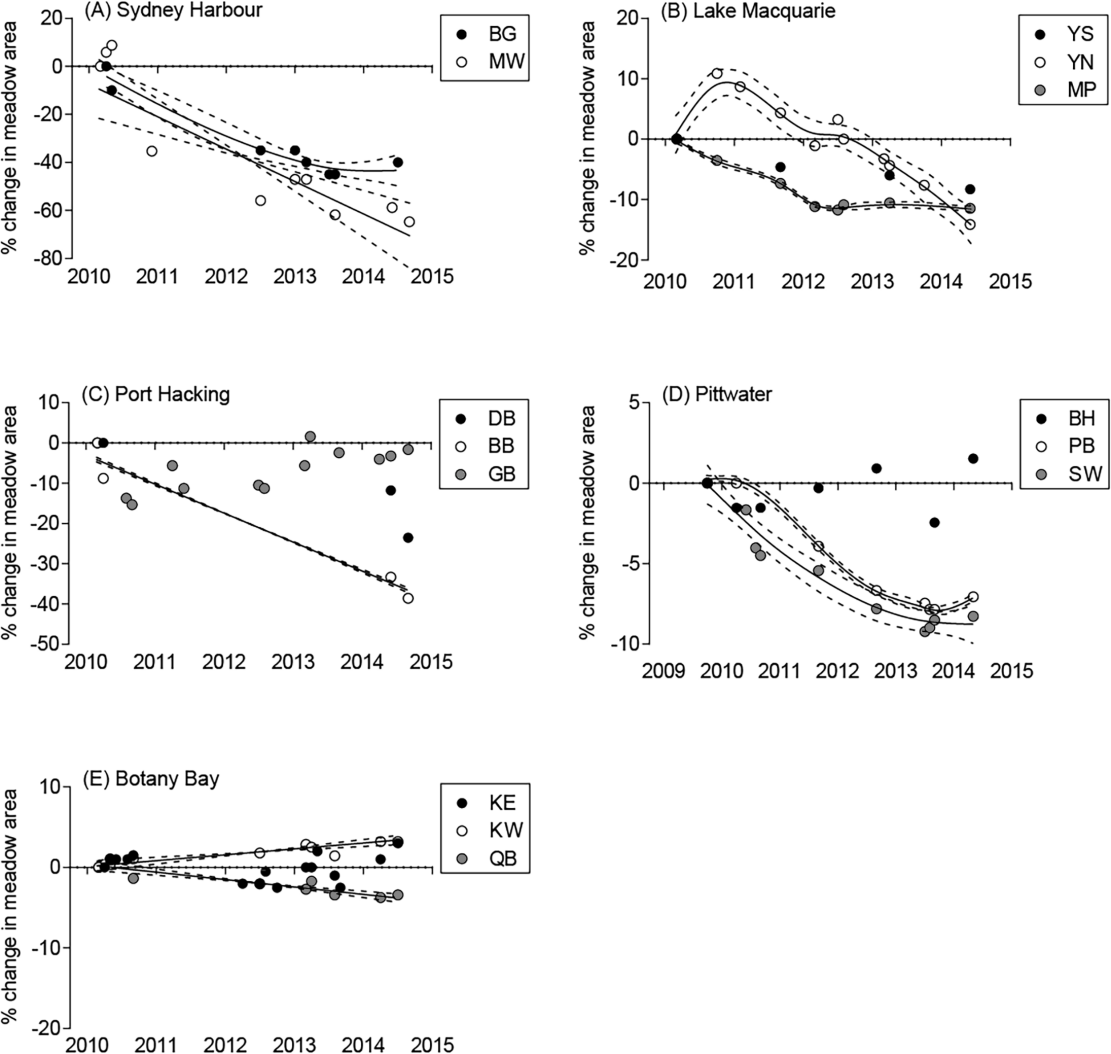
Temporal percent change in *P*. *australis* meadow cover from late 2009/early 2010 to mid-2014 in all sites within five estuaries. Each data point represents total percent change since the initial time point. Thin-plate spline regression lines (and 95% confidence interval) are added for those sites where change was significant over the study period. YS = Yacht Club South, YN = Yacht Club North, MP = Marks Point, BH = Barrenjoey Head, PB = Palm Beach Ferry, SW = Seaplane Wharf, BG = Balgowlah, MW = Manly Wharf, KE = Kurnell East, KW = Kurnell West, QB = Quibray Bay, DB = Dolans Bay, BB = Burraneer Bay GB = Gunnamatta Bay.

*Posidonia australis* meadows declined by 2–40% total area at 11 of 14 study sites (Fig 4; F_4,13_ = 14.77; P = 0.001). This equates to a total loss of 7.61 ha, or 21.2% of all seagrass meadow in the study area, between October 2009 and September 2014. The greatest declines were observed in Sydney Harbour, in which 40% of the total meadow area (1.12 ha) was lost between March 2010 and September 2014 (Fig 4). Estimates of change in seagrass cover over time reveal that both Manly Wharf and Balgowlah are losing seagrass habitat at an average rate of more than 10% per year. Specifically, 7006 m^2^ (36.6%) was lost from Balgowlah, while 4146 m^2^ (46.1%) was lost from Manly Wharf (Table 1). In total, the perimeter of both meadows decreased by 3476 m. The number of distinct seagrass patches at Balgowlah declined from nine to six, while Manly Wharf increased from 23 to 31 patches (Table 1) as a result of further fragmentation within existing patches. The greatest declines in Sydney Harbour appear to have occurred between 2010 and 2012 (Fig 5A).

**Table 1:** Site locations and total change in meadow area, perimeter and patchiness from the first available image (late 2009/early 2010), until the last available image (mid-2014). Study sites were ~520,000m^2^. Declines in seagrass area are highlighted in bold. (continued below).

Estuary	Site	Latitude	Longitude	Area change (m^2^)
Lake Macquarie	Yacht Club North	-33.03879	151.65541	**- 8934.7**
	Yacht Club South	-33.04111	151.65477	**- 13362.5**
	Marks Point	-33.05220	151.64171	**- 9914.4**
Pittwater	Barrenjoey Head	-33.58196	151.32306	+ 4442.4
	Seaplane Wharf	-33.58693	151.32311	**- 10312.0**
	Palm Beach Ferry	-33.59517	151.31835	**- 15826.1**
Sydney Harbour	Manly Wharf	-33.80145	151.28541	**- 7006.1**
	Balgowlah	-33.80436	151.27131	**- 4145.5**
Botany Bay	Quibray Bay	-34. 00405	151.17550	**- 3666.8**
	Kurnell West	-34.00590	151.18955	+ 1162.1
	Kurnell East	-34.00462	151.21474	+ 823.4
Port Hacking	Dolans Bay	-34.06742	151.13022	**- 1160.6**
	Burraneer Bay	-34.06746	151.13428	**- 8118.5**
	Gunnamatta Bay	-34.07226	151.14565	**- 121.4**
Perimeter change (m)	Patches '09/10	Patches '14		
- 6339.0	40	12		
+ 1867.4	70	66		
+ 3786.9	53	63		
+ 1881.8	12	2		
+ 13015.9	65	80		
+ 3008.3	44	47		
- 2345.1	23	31		
- 1131.2	9	6		
- 6770.7	313	258		
- 1035.4	196	202		
+ 760.4	42	28		
+ 140.1	1	2		
- 6718.1	129	28		
- 5991.2	209	154		

The three sites within Lake Macquarie had meadow losses ranging from 5.4–17.1%, equating to a total loss in area of 3.2 ha. There were prominent declines along the western edges of the meadows (furthest from the shore) where bare patches began to merge. This is particularly evident at Yacht Club North, where the total meadow perimeter decreased by 6339 m, along with a drop in the number of patches from 40 to 12 (Table 1). Losses at both Yacht Club South and Marks Point coincide with perimeter increases (Table 1), which appear to be the result of expansion of bare patches. Estimates of temporal change in area revealed relatively consistent declines across all three meadows, of 2–5% per year (Fig 5B).

The declines in Port Hacking ranged from 0.2–27.5% since early 2010, equating to a total of 0.94 ha. The greatest meadow loss was within Burraneer Bay (- 0.81 ha), which also shows the most rapid rate of decline, estimated at over 7% yr^-1^ (Fig 5C). This site appears mostly unaffected by boat moorings and propeller scars, and instead suggests evidence of increased sedimentation within the meadow; with the number of patches rapidly decreasing from 129 in 2010 to just 28 in 2014. Overall, Dolans Bay decreased in area by 0.12 ha, with the most rapid decline occurring in 2014 (Fig 5C). Dolans Bay was the smallest meadow of the study, with an area of just 0.92 ha in March 2010. While the area existed as a single intact ‘patch’ in the early imagery, there was evidence of propeller scarring within the meadow adjacent to private jetties, which resulted in the meadow fragmenting into two patches by 2014. Gunnamatta Bay recorded a total loss in area of 0.2%, however the pattern of decline was temporally inconsistent and appears to be gradually increasing following an initial decline in 2010 (Fig 5C).

The total decline in Pittwater was 3.8% (2.2 ha), however, there were stark contrasts among the three study sites. The Barrenjoey Head meadow experienced natural expansion at its western edge in 2014, increasing by 2.5% (0.4 ha) of its area since 2009, even with the expansion of clearings from boat moorings within the meadow (Fig 5D). There were significant declines of 4.1% at Seaplane Wharf and 10.9% at Palm Beach Ferry (a total of 2.6 ha). Seaplane Wharf and Palm Beach Ferry meadows declined at relatively constant rates, with the most rapid declines occurring between 2010 and 2013 (Fig 5B). All three sites increased in perimeter, with the two declining meadows, Seaplane Wharf and Palm Beach Ferry also showing an increase in the number of patches as a result of meadow fragmentation.

Lastly, the total decline of *P*. *australis* in Botany Bay was small compared to other estuaries, at 0.17 ha or just under 1% of total meadow area. Significant declines were recorded at only one site, Quibray Bay, which declined by 0.37 ha between 2010 and 2014. Kurnell West and Kurnell East both increased in total area, although the change was minor (both increases <1%; Fig 5D; Table 1). Kurnell East experienced a wide variation in percent cover over the five year time span. While extensive losses became apparent in 2010 as a result of construction, meadow expansion at the edges (e.g. Fig 3) reduced the overall rate of decline.

## Discussion

Our analysis of high resolution aerial photography demonstrates that seagrass meadows dominated by *Posidonia australis* underwent consistent declines in southeastern Australia between 2009–14, with a maximum estimate of 40% loss of habitat across five endangered estuaries. These declines are alarmingly rapid, occurring over a very short time-frame (five years between 2009–2014). In the most extreme case, our study estimates that meadows of *P*. *australis* in Sydney Harbour are declining at an average rate greater than 10% per year, exceeding the global rate of seagrass decline [7]. The *Posidonia australis* meadows in the five estuaries represented in this study were declared as endangered under the New South Wales Fisheries Management Act (1994) in 2010 and by the Environment Protection and Biodiversity Conservation Act (1999) in 2015. This was largely based on declines of 12–57% in area between the 1940s to 1980s [22,29]. Considering our observations of declines between 1–40% in area have occurred since the species has been listed as endangered, the future of these meadows is of immediate concern.

Although *P*. *australis* can recolonise areas following natural disturbances (e.g. regrowth from existing rhizome mat; [46]), numerous examples show that *P*. *australis* has either never returned, or has only recolonised at very slow rates following anthropogenic disturbances [40]. While there is evidence of vegetative growth along the edges of some meadows (Barrenjoey Head in Pittwater and Kurnell in Botany Bay), it appears to be very slow (< 1% increase yr^-1^), while the expansion of bare sandy patches created by anthropogenic disturbance can be immediate. An example of the persistence of disturbed clearings in *P*. *australis* meadows are the multiple 20 m wide circular clearings created from seismic blasting at Jervis Bay NSW in 1962 [40]. Monitoring of these bare patches after 27 years revealed negligible signs of recolonisation [40]. Further studies have reported slow but consistent recovery after 38 years [37], but remnants of these bare patches are still clearly visible in Nearmap imagery from August 2014, 52 years after the damage occurred.

The most alarming declines of *P*. *australis* over the past five years have occurred within Sydney Harbour at Balgowlah (a loss of 46.1%) and Manly Wharf (a loss of 36.6%). At both sites, bare sand ‘blowouts’ from boat moorings created substantial meadow fragmentation, which resulted in the rapid decline of remaining seagrass patches. This is assumed to be primarily the result of sediment destabilisation caused by boat mooring chains scouring the sediment with each wind direction change [15,25]. Boat moorings causing damage to seagrass beds (particularly *P*. *australis*) and have not gone unnoticed by government authorities [38]. At Manly Wharf alone, thirty ‘seagrass-friendly’ moorings were installed in 2009 to replace some traditional block and chain moorings [47]. While an engineering solution to avoid scouring the seabed is a good initiative, it is expected that seagrass-friendly moorings will be of maximum benefit within meadows that are not already in a state of decline. Within *P*. *australis* meadows, the damage caused by mooring blowouts results in a self-perpetuating cycle of decline, in which the seagrass cannot recolonise within the destabilised sediment [33] and runaway fragmentation ensues regardless of moorings removal [25].

Similar impacts from boating infrastructure and activity were evident within meadows of *P*. *australis* in Lake Macquarie and Pittwater. Since early 2010, significant decreases in meadow area were combined with increases in meadow perimeter; a result of the continued expansion of bare patches within meadows, caused by moorings. We estimated a decline of 3.2 ha of seagrass across the three sites within Lake Macquarie in this study, consistent with the recent estimate by Wright et al. ([48]) and Glasby and West ([38]) that 6–7 ha of *P*. *australis* had been cleared from the entirety of Lake Macquarie by boat moorings. While boat moorings were also a major contributor to declines within Pittwater, there was evidence of additional removal via propeller damage, particularly within the meadow at Seaplane Wharf, which lost a total of 4.1% (~1 ha). Scouring from propellers removes seagrass leaves and rhizomes from narrow strips (up to 50 cm) that are often hundreds of metres in length [25]. These tracks often become natural channels for incoming and outgoing tides, preventing seagrass from recolonising within the tracks and accelerating meadow decline [15].

In Port Hacking, we recorded a 27.5% decline in one site (Burraneer Bay) since early 2010, which appears to be the result of sediment movement within the estuary. The rapid accumulation of sediment within a meadow can smother seagrass shoots [15,32,49]. While the total volume of sediment within Port Hacking is thought to remain relatively constant, there is a continuous redistribution of sediments within the estuary as it attempts to reach equilibrium following numerous anthropogenic disturbances since the 1900s [42]. Increased sediment influxes can also be the result of dredging, flooding and poor catchment management following land clearing [15], and have been known to smother seagrasses in Hervey Bay [32] and Moreton Bay [49] in north-eastern Australia.

While declines were observed in all five estuaries, there were three sites that experienced slight increases in meadow area over five years. Our analysis suggested meadow expansions of 0.2 ha in Botany Bay (Kurnell East and Kurnell West) and 0.4 ha in Pittwater (Barrenjoey Head). The apparent meadow expansion at Barrenjoey Head in Pittwater was estimated at approximately 0.5% growth (0.08 ha) per year. This is a positive sign that may be a result of relatively few boat moorings in this location. However, this growth rate exceeds the expected horizontal growth rates of P. australis in this region [37], and further ground-truthing is required to confirm that the observed expansions are in fact a result of solely P. australis expansion. While this growth may seem promising, it is not to say that these meadows were stable or free from anthropogenic disturbance. In Kurnell East (Silver Beach), approximately 180 m^2^ of *P*. *australis* was reported lost in July 2010, following the laying of submarine power cables. The loss of seagrass directly resulting from this damage is more than 2290 m^2^ as of September 2014. This impact has been acknowledged [50] and a restoration program is currently underway (in early 2017) to re-establish the damaged meadow following project completion.

This research shows that within already heavily-impacted meadows in southeastern Australia, declines of *P*. *australis* are showing no signs of slowing, regardless of improved water quality conditions or initiatives such as the introduction of seagrass-friendly boat moorings [47]. Without a more concerted approach it is unlikely that the resources already directed to legislative protection and direct actions will provide a sound return in the form of recovery of meadow condition. While further damage of these seagrass meadows could be reduced by severe limitations to human activities (e.g. boating, dredging, dumping) within these areas, this is obviously a logistically difficult solution to implement in such urbanised estuaries. It is imperative, however, that compromises are made to improve the planning and management of human activities that will impact seagrass meadows. Moreover, the development of novel transplantation methods to restore damaged *P*. *australis* meadows is also becoming increasingly urgent [38,51].

The results presented here show the usefulness of high resolution aerial imagery to quickly and easily monitor the conditions of meadows on small spatial and temporal scales. These methods could be readily applied to other species or habitats including seagrasses [52], mangroves [53] and wetland [54] or arid zone vegetation [55]–all of which are often mapped at coarser spatial and temporal scales. Aerial imagery databases do, however, carry limitations in image availability, quality and resolution. In the present study we were restricted to studying only regions where suitable images were available, and the images selected for analysis were constrained by image quality and water quality. Additionally, ground-truthing is an integral part of any remote sensing data-set, to confirm the accuracy of species presence and absence at the appropriate scale of observation. However, it is often unfeasible to confirm all areas of change from in situ ground-truthing, whether due to logistical constraints, low-accuracy or expensive geolocation (especially in sub-tidal studies as GPS does not operate through water), and a lack of historical ground-truthing to accompany archived images. This study demonstrated that inferences about habitat condition and the rate and scale of change are still achievable based on the information stored in aerial image databases.

While the spatial extent of *P*. *australis* within New South Wales has been systematically mapped at broad scales [29], the techniques used in those surveys are unable to take account of discontinuities within meadows, such as ‘holes’ and fragmentation caused by human activities. Here, we show that the use of high resolution, commercially available aerial imagery can accurately quantify the extent of within-meadow damage and decline at relatively small spatial scales. Ultimately, this kind of information is essential to improve the monitoring of both seagrass meadows and continued anthropogenic impacts.

## Supporting information

S1 Fig **Map of Pittwater, New South Wales (A), with visual representation of change in meadow area (B) from three selected sites within the estuary from 2010 to 2014.** Inlaid boxes represent enlarged sections from Barrenjoey Head (C), Seaplane Wharf (D), and Palm Beach Ferry (E). Aerial imagery reprinted under a CC BY license with permission from Nearmap, original copyright 2014.
(TIF)

S2 Fig Georectified Nearmap imagery representing declines attributed to fragmentation from boat moorings at site ‘Manly Wharf’ in Sydney Harbour. Top image was taken in Nov 2009; bottom image was taken in Sep 2014. Aerial imagery reprinted under a CC BY license with permission from Nearmap, original copyright 2014.
(PNG)

S3 Fig Georectified Nearmap imagery showing the impact of power cable burial in Botany Bay after four years. Clockwise from top left: Intact meadow in Apr 2010; initial construction first seen in Aug 2010; distinct damage left from cabling in Jul 12; latest available imagery shows negligible recolonisation in Sep 14. Aerial images reprinted under a CC BY license with permission from Nearmap, original copyright 2014.
(TIF)

S4 Fig Georectified Nearmap imagery showing apparent causes of *P*. *australis* decline. The top panel shows a *P*. *australis* meadow in Port Hacking (Burraneer Bay site) in Jun 2010 (left) and Sep 2014 (right) with evidence of increased sedimentation/sediment movement within the estuary, potentially causing shoot burial. The bottom panel shows a *P*. *australis* meadow in Pittwater (Seaplane Wharf site) in Oct 2009 (left) and Oct 2014 (right) showing distinct lines caused by propeller damage. Aerial imagery reprinted under a CC BY license with permission from Nearmap, original copyright 2014.
(PNG)

S5 Fig Cover of seagrass at each study site quantified during in-situ ground-truthing. (PNG)
