# Correction: Seagrass on the brink: Decline of threatened seagrass *Posidonia australis* continues following protection

**DOI:** 10.1371/journal.pone.0216107

**Published:** 2019-04-23

**Authors:** Suzanna M. Evans, Kingsley J. Griffin, Ray A. J. Blick, Alistair G. B. Poore, Adriana Vergés

In the Abstract, there is an error in the ninth sentence. The correct sentence is: Our results demonstrate that seagrass meadows dominated by *P*. *australis* underwent declines of ~ 0.2–40% total area at 11 out of 14 study sites between 2009 and 2014.
