# Correction: Correction: Seagrass on the brink: Decline of threatened seagrass *Posidonia australis* continues following protection

**DOI:** 10.1371/journal.pone.0271005

**Published:** 2022-06-30

**Authors:** Suzanna M. Evans, Kingsley J. Griffin, Ray A. J. Blick, Alistair G. B. Poore, Adriana Vergés

There is an error in the Correction published on April 23, 2019. The Correction should have also included the following text:

“In the Results subsection of the Methods, there is an error in the first sentence. The correct sentence is: *Posidonia australis* meadows declined by 0.2–40% total area at 11 of 14 study sites (Fig 4; F_4,13_ = 14.77; P = 0.001).”

## References

[1] Evans SM, Griffin KJ, Blick RAJ, Poore AGB, Vergés A (2019) Correction: Seagrass on the brink: Decline of threatened seagrass *Posidonia australis* continues following protection. PLoS ONE 14(4): e0216107. 10.1371/journal.pone.0216107 31013329 PMC6478339

