# Correction: Correction: Correction: Seagrass on the brink: Decline of threatened seagrass *Posidonia australis* continues following protection

**DOI:** 10.1371/journal.pone.0292583

**Published:** 2023-10-04

**Authors:** The PLOS ONE Staff

## Notice of Republication

This article was republished on September 15, 2023, to correct an error in the fourth sentence of the Correction notice. The publisher apologizes for the error. Please download this article again to view the correct version. The originally published, uncorrected article and the republished, corrected article are provided here for reference.

## Supporting information

S1 File Originally published, uncorrected article. (PDF)

S2 File Republished, corrected article. (PDF)
