# Supplementary figures and images for: Seagrass on the brink: Decline of threatened seagrass Posidonia australis continues following protection
Source: PLoS One. 2018 Apr 6;13(4):e0190370. doi: 10.1371/journal.pone.0190370 (PMC5889071; doi:10.1371/journal.pone.0190370)

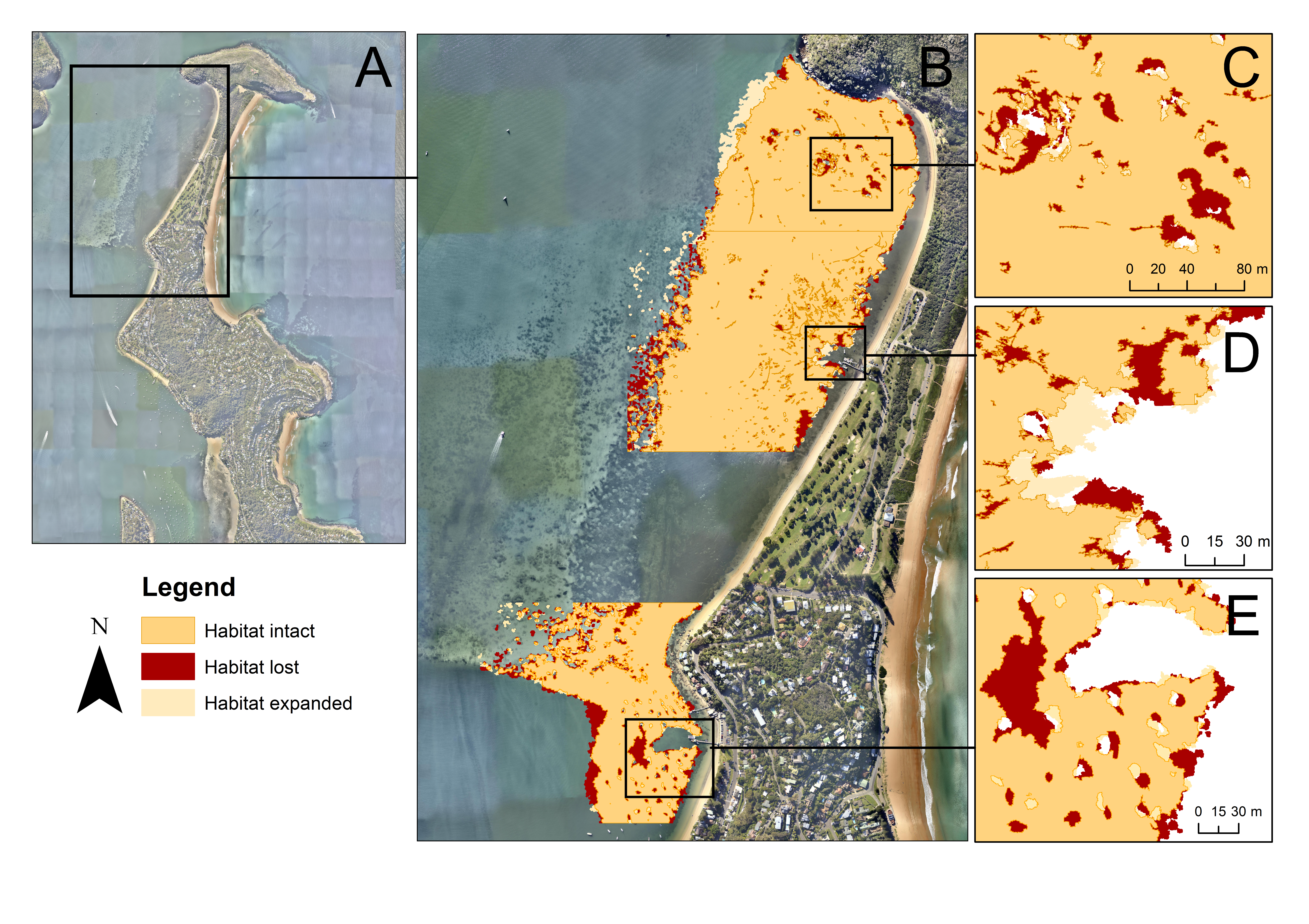

Supplement: S1 Fig — Map of Pittwater, New South Wales (A), with visual representation of change in meadow area (B) from three selected sites within the estuary from 2010 to 2014. Inlaid boxes represent enlarged sections from Barrenjoey Head (C), Seaplane Wharf (D), and Palm Beach Ferry (E). Aerial imagery reprinted under a CC BY license with permission from Nearmap, original copyright 2014. (TIF) [file pone.0190370.s001.tif]

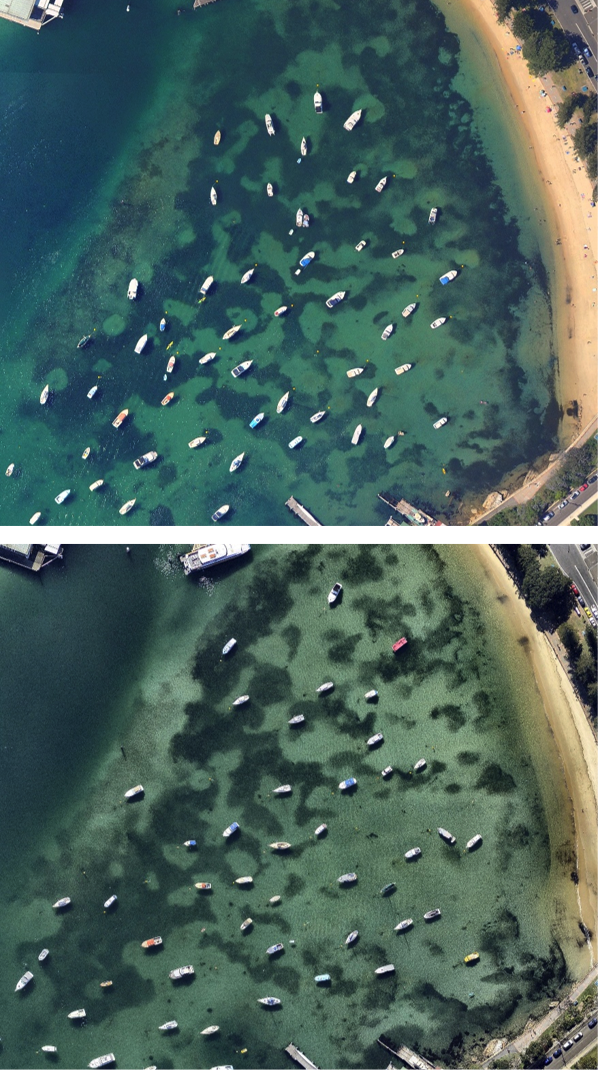

Supplement: S2 Fig — Top image was taken in Nov 2009; bottom image was taken in Sep 2014. Aerial imagery reprinted under a CC BY license with permission from Nearmap, original copyright 2014. (PNG) [file pone.0190370.s002.png]

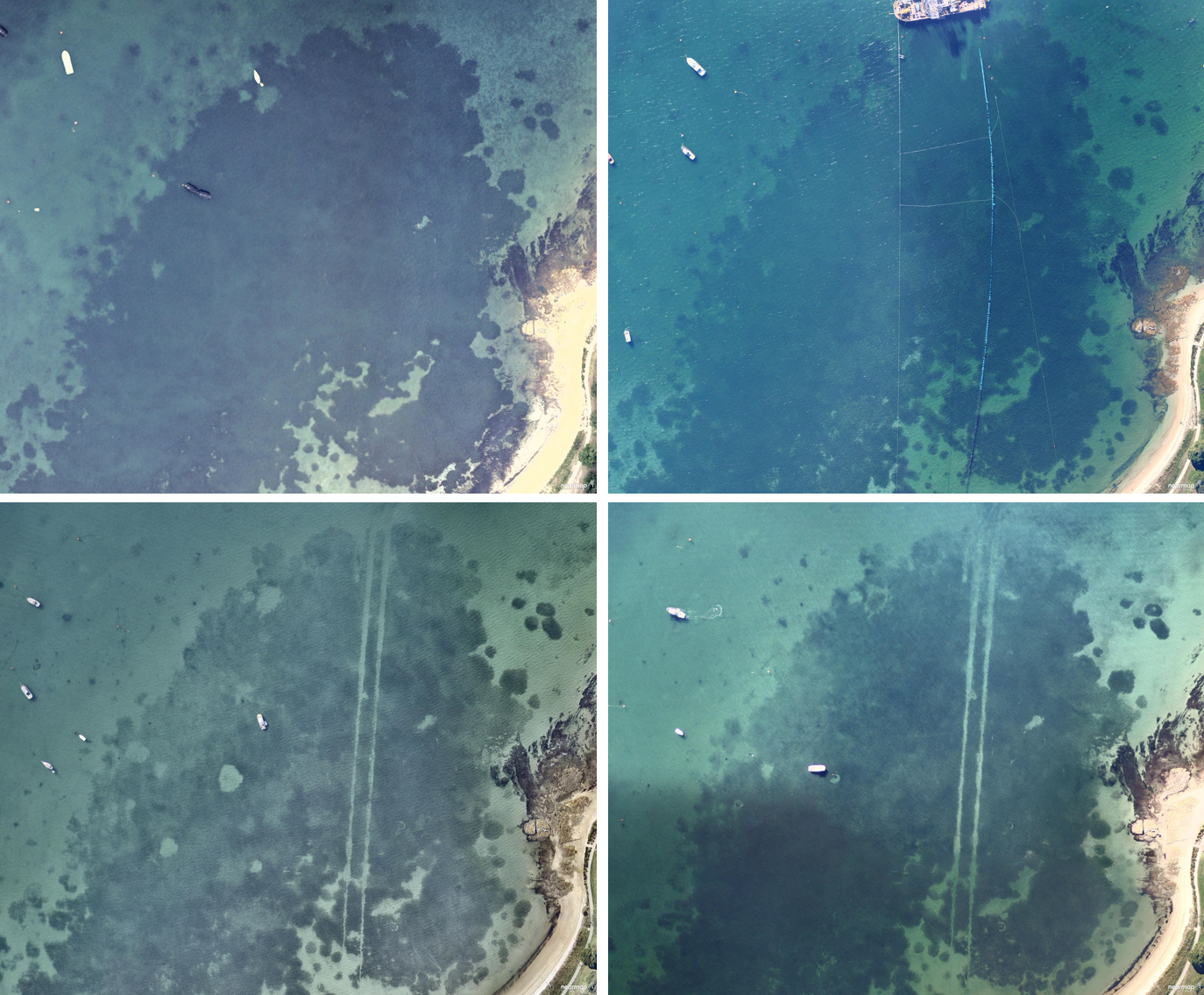

Supplement: S3 Fig — Clockwise from top left: Intact meadow in Apr 2010; initial construction first seen in Aug 2010; distinct damage left from cabling in Jul 12; latest available imagery shows negligible recolonisation in Sep 14. Aerial images reprinted under a CC BY license with permission from Nearmap, original copyright 2014. (TIF) [file pone.0190370.s003.tif]

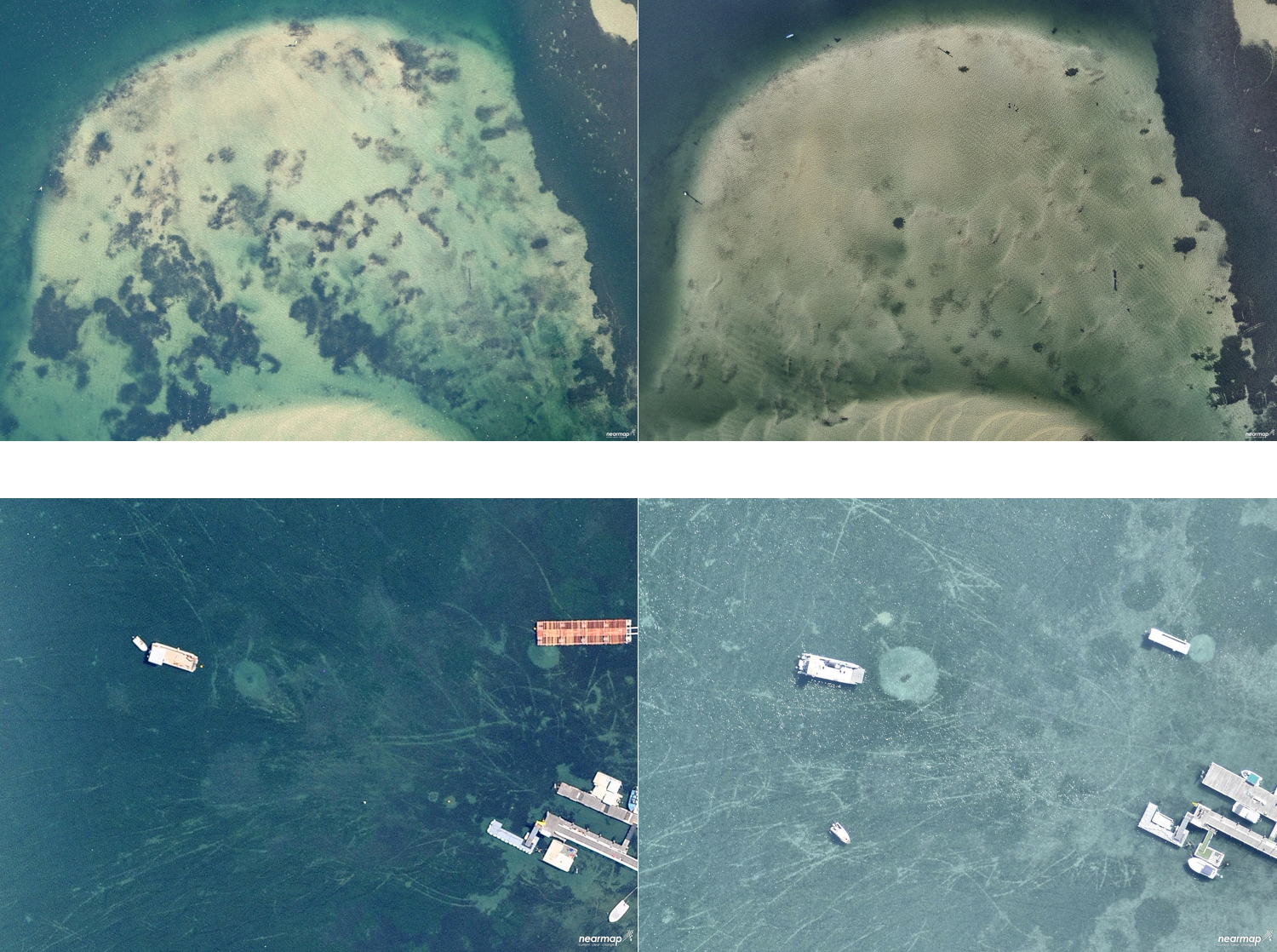

Supplement: S4 Fig — The top panel shows a P. australis meadow in Port Hacking (Burraneer Bay site) in Jun 2010 (left) and Sep 2014 (right) with evidence of increased sedimentation/sediment movement within the estuary, potentially causing shoot burial. The bottom panel shows a P. australis meadow in Pittwater (Seaplane Wharf site) in Oct 2009 (left) and Oct 2014 (right) showing distinct lines caused by propeller damage. Aerial imagery reprinted under a CC BY license with permission from Nearmap, original copyright 2014. (PNG) [file pone.0190370.s004.png]

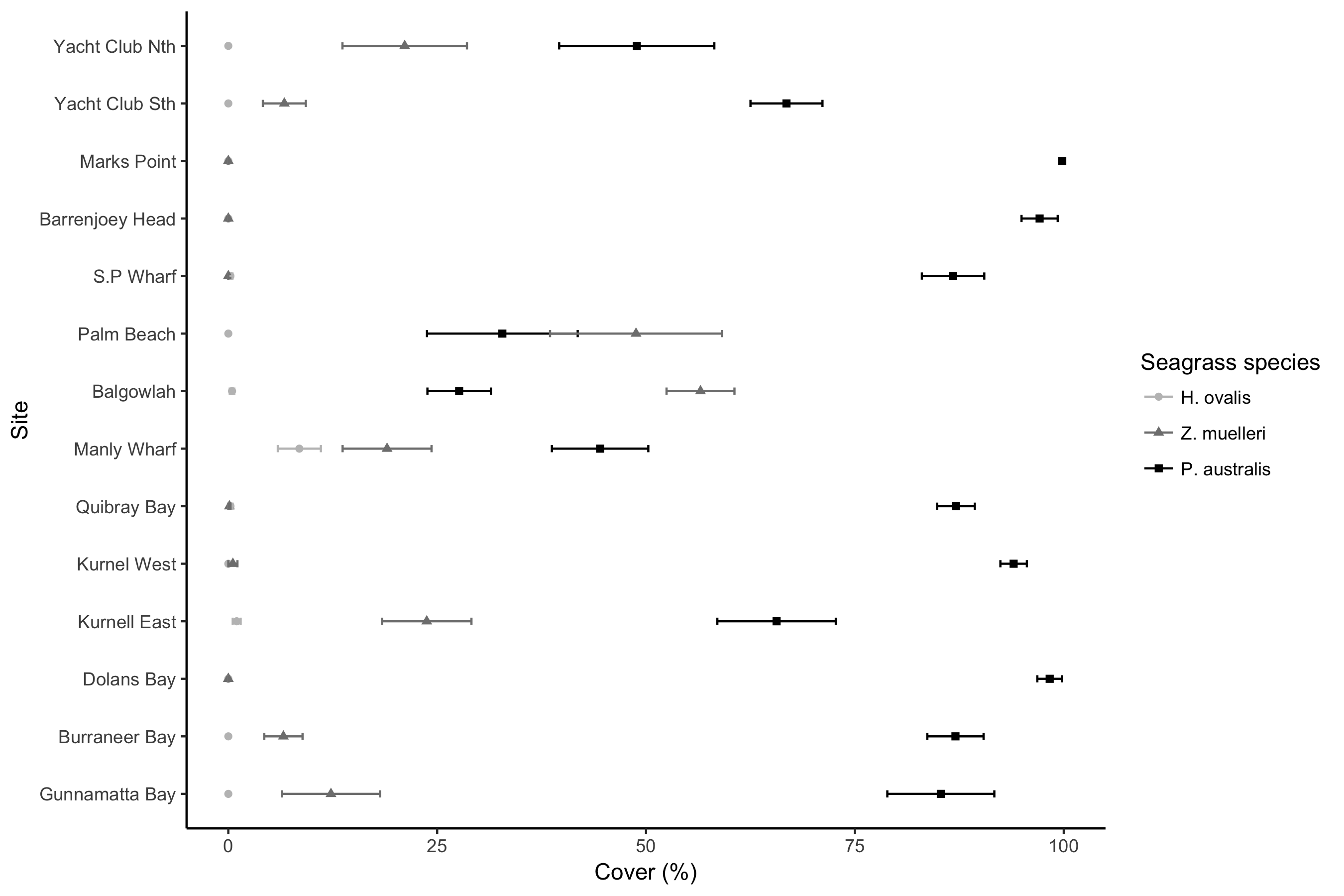

Supplement: S5 Fig — (PNG) [file pone.0190370.s005.png]
