# Supplementary material for: Correction: Correction: Correction: Seagrass on the brink: Decline of threatened seagrass Posidonia australis continues following protection
Source: PLoS One. 2023 Oct 4;18(10):e0292583. doi: 10.1371/journal.pone.0292583 (PMC10550118; doi:10.1371/journal.pone.0292583)
Supplement: S2 File — (PDF) [file pone.0292583.s002.pdf]

PLOS ONE

CORRECTION

Correction: Correction: Seagrass on the brink: Decline of threatened seagrass *Posidonia australis* continues following protection

Suzanna M. Evans, Kingsley J. Griffin, Ray A. J. Blick, Alistair G. B. Poore, Adriana Vergés

There is an error in the Correction published on April 23, 2019. The Correction should have also included the following text:

"In the Results subsection of the Methods, there is an error in the first sentence. The correct sentence is: *Posidonia australis* meadows declined by 0.2–40% total area at 11 of 14 study sites (Fig 4; $F_{4,13} = 14.77$; $P = 0.001$)."

Reference

1. Evans SM, Griffin KJ, Blick RAJ, Poore AGB, Vergés A (2019) Correction: Seagrass on the brink: Decline of threatened seagrass *Posidonia australis* continues following protection. PLoS ONE 14(4): e0216107. https://doi.org/10.1371/journal.pone.0216107 PMID: 31013329

Check for updates

OPEN ACCESS

Citation: Evans SM, Griffin KJ, Blick RAJ, Poore AGB, Vergés A (2022) Correction: Correction: Seagrass on the brink: Decline of threatened seagrass *Posidonia australis* continues following protection. PLoS ONE 17(6): e0271005. https://doi.org/10.1371/journal.pone.0271005

Published: June 30, 2022

Copyright: © 2022 Evans et al. This is an open access article distributed under the terms of the Creative Commons Attribution License, which permits unrestricted use, distribution, and reproduction in any medium, provided the original author and source are credited.
